# Was It Cocaine?

**Published:** 1888-06

**Authors:** R. H. Harrison

**Affiliations:** Columbus, Tex.


### WAS IT THE COCAINE?

*By R. H. Harrison, Jr., M. D., Columbus, Tex.*

For Daniel's Texas Medical Journal.

I WAS called, a few days since, to see the following case, and as it presents some interesting features, report it:

Mrs. S., aged about thirty, of fair complexion, with black hair and eyes, and of a decidedly nervous temperament, has been suffering for some time past with odontalgia. She is tall and well formed, and three months ago passed through her fourth confinement without any unusual trouble.

A dentist, Dr. Z., being called in to extract the carious teeth, and finding her very nervous, gave her a whiskey toddy, followed by three hypodermic injections into the gum of a 6 per cent. solution of Cocaine Mur. Dr. Z. states that she *fainted* immediately after the injections. I arrived in the course of fifteen or twenty minutes after the injections, and found her in the following condition:

Insensible, pupils widely dilated, jaws locked, head drawn back, the extensors of the arms contracted and hands clenched, feet extended and incurved, breathing very rapid (about 40 per minute), superficial and spasmodic, pulse 80, and rather hard, surface cold.

I had her inhale aromatic spirits of ammonia, and the extremities rubbed vigorously with mustard. In a few minutes her respiration was fuller and easier, the extremities relaxed, but the trismus and insensibility remained. In ten minutes she had another convulsion, in which the opisthotonos was well marked, the spasm lasting about ten minutes altogether. The same treatment was administered as in the preceding convulsion, but apparently without result. This convulsion had barely passed off, when another came on of a like severity and duration. After this, the third convulsion, the trismus was less marked, and with some difficulty she swallowed a half drahm each of F. Ex. Lobelia an Tr. Assafœtida, (the only antispasmodmic at hand). She had one convulsion about twenty minutes after this, but it was much milder than the preceding ones. After this she was thoroughly relaxed and very prostrate, her only complaint being of soreness throughout the muscular system, and a sense of weakness. She had no further treatment, except a liniment for the soreness, and her recovery was prompt.

It appears that cocaine is capable of producing tetanic convulsions, when administered in large doses; but the short space of time that elapsed between the administration and the onset of the convulsions, would leave the question an open one. I may add also, that the surface temperature was mever raised above normal, and that she had no inclination to sleep for several hours after the convulsions had ceased.

I would be glad to see something from some of the profession who have had experience with the drug.

